# Renal long COVID-19: an ongoing debate requiring robust evidence

**DOI:** 10.1590/2175-8239-JBN-2025-E001en

**Published:** 2025-02-24

**Authors:** Heitor S. Ribeiro, Emmanuel A. Burdmann, Luis Yu

**Affiliations:** 1 Universidade de São Paulo, Faculdade de Medicina, Hospital das Clínicas HCFMUSP, Laboratório de Investigação Médica (LIM 12), São Paulo, SP, Brazil.

COVID-19 has a devastating impact on both pulmonary and extrapulmonary systems. The kidneys are frequently affected during the acute phase of COVID-19, with approximately 30% of patients developing acute kidney injury (AKI) and nearly half ultimately dying^1^. For survivors of COVID-19 hospitalization, long-term follow-up and multi-organ screening have been recommended due to the impact on long-term morbidity and mortality and because of the potential burden of long COVID-19 for healthcare systems^2^. Most of the available evidence on long COVID-19 has been focused on cognitive, neurological, psychological, and pulmonary outcomes^3^. Few prospective cohort studies on renal long COVID-19 have been published, as detailed in Table 1.

**Table 1:** Prospective cohort studies investigating renal long COVID

Author and year	Sample (n and characteristics)	Kidney function markers and time points	AKI during hospital stay	Kidney function outcomes at follow-up	CKD development and/or progression
Morin et al.^4^ 2021	478 hospitalized patients	sCr 4 months	20% (KDIGO)	2/177 patients displayed a persistent alteration of kidney function	NR
Li et al.^5^ 2022	155 hospitalized patients	sCr 3, 12 and 24 months proteinuria 24 months	NR	eGFR declined from 3 to 12 months, but not from 12 to 24 months Proteinuria 7% at 24 months	NR
Gu et al.^6^ 2022	1734 hospitalized patients	eGFR, proteinuria, and hematuria 6 and 12 months	6% (KDIGO)	survivors with AKI had a higher kidney function reduction	NR
Huang et al.^7^ 2023	1733 hospitalized patients	eGFR 6 months	6% (KDIGO)	eGFR < 90 mL/min/1.73 m^2^: 479/1378 (35%)	NR
Schmidt-Lauber et al.^8^ 2023	443 non-severe cases	eGFR, albuminuria, Dickkopf3, hematuria and pyuria 9 months	non-severe cases (no AKI)	mean eGFR mildly lower in post-COVID-19 than non-COVID-19 subjects	CKD development and albuminuria did not differ between post-COVID-19 and non-COVID-19
Mehrotra-Varma et al.^9^ 2024	748 hospitalized and non-hospitalized patients with type 1 diabetes	ICD-10 diagnostic codes for CKD	NR	NR	Compared to non-COVID-19 patients, both hospitalized (11.4% vs. 1.1%) and non-hospitalized (7.7% vs. 1.1%) patients were more likely to develop CKD
Ribeiro et al.^10^ 2024	655 hospitalized patients	eGFR, albuminuria, abnormal urine sediment 7 months	79%: 35% community-acquired and 43% hospital-acquired (KDIGO)	16% developed incident low eGFR (<60 mL/min/1.73m^2^) 27% had an eGFR decline of ≥25% 30% had albuminuria and 20% had abnormal urine sediment	

Abbreviations – AKI: Acute Kidney Injury; CKD: chronic kidney disease; eGFR: estimated glomerular filtration rate; ICD-10: International Classification of Diseases, Tenth Revision; KDIGO: Kidney Disease Improving Global Outcomes; KRT: kidney replacement therapy; MAKE: major adverse kidney events; NR: not reported; sCr: serum creatinine.

In this issue of the Brazilian Journal of Nephrology, de Andrade et al.^11^ prospectively evaluated 360 critically ill COVID-19 patients with AKI during the acute phase of the disease that required a consultation with a nephrologist in a tertiary hospital in the city of Porto Alegre, RS. The authors defined AKI according to the Kidney Disease: Improving Global Outcomes (KDIGO) criteria based on serum creatine (sCr). They also evaluated albuminuria and kidney replacement therapy (KRT) need. Recovery of kidney function was defined as independence from KRT or sCr returning to within 50% of baseline, according to the Acute Dialysis Quality Initiative. Overall, they found a very high frequency of severe AKI (KDIGO III - 90.8%), in which 90% required KRT, resulting in an elevated 90-day mortality rate (88%) and only 10% patients recovered renal function. After one year of follow-up, mortality rate was also very high (89%). Among survivors, the estimated glomerular filtration rate (eGFR) after one year was lower than the baseline levels. One methodological issue of this study was the proposed definition of baseline sCr for diagnosing AKI, in which authors looked at the mean creatinine value between 7 and 365 days before hospitalization. However, if not available, the minimum sCr value during hospitalization was adopted. This variation in the definition may bias the internal validity of the AKI diagnosis, especially because the authors do not inform the frequency of patients without baseline sCr. By adopting the minimum sCr value during hospitalization, authors may have overestimated AKI diagnosis. Furthermore, loss of muscle mass, commonly observed in critically ill patients with COVID-19, may have led to a decrease in sCr levels during hospitalization^12^, which may have resulted in lower sCr value than the actual baseline.

Another finding that warrants consideration is the extremely high frequency of severe AKI, seldom reported in the literature. Corroborating this finding, 98% of patients needed mechanical ventilation and most of them required KRT (90%). As expected, a nephrology consultation in the critical setting is usually requested for the more severe cases, especially in a pandemic situation, resulting in the selection of severe AKI cases. Nevertheless, it has been reported that nephrology consultation for COVID-19 patients usually results in higher patient survival^13,14^. Therefore, there was a clear sample selection bias, which limits generalization to non-severe COVID-19 patients and patients with earlier stages of AKI who are not usually referred to nephrologists.

The primary endpoints were 90-day mortality, kidney function recovery, and KRT dependence. However, the authors did not report the median survival time. At the end of the 90-day period, only 12% of the patients had survived. The assumption of a 90-day follow-up period may lead to an expectation of a long-term follow-up after hospital discharge, which did not happen for most of the patients. Indeed, mortality at 30 days was 66% and at 60 days 85%, similar to 90-day mortality rate. Therefore, these findings should be carefully interpreted as an in-hospital outcome. In addition, authors included a one-year follow-up for a very small sample of survivors (n = 43; 12%), in which only 25 of the remaining patients (58%) had available eGFR measurement. They have demonstrated that the survivors’ eGFR was lower than the baseline eGFR (65.9 vs 85.5 mL/min/1.73 m^2^). Although the analysis was secondary and exploratory, caution should be taken in extrapolating the findings to COVID-19 survivors, as the study sample was derived from a biased cohort with very high in-hospital mortality, resulting in long follow-up of a very small sample.

Although the study is novel in recruiting a sample of patients who required a nephrology consultation, there is already enough data in the literature demonstrating the impact of COVID-19 on kidney-related outcomes during the in-hospital period^1^. At the present time, the discussion should focus on whether long COVID-19 affects the kidneys, rather than how the kidneys are a target organ during the acute phase of infection. As demonstrated in Table 1, there are currently few available studies addressing this important issue. Our research group is conducting a prospective cohort study with over 650 survivors of COVID-19 hospitalization in the city of São Paulo, SP^15^, with annual follow-up for up to five years after hospital discharge. Preliminary data presented at the XXXII Brazilian Congress of Nephrology and the ASN Kidney Week 2024 showed that 27% of patients experienced a decline in eGFR ≥25%, and 16% were incident for low eGFR (<60 mL/min/1.73 m^2^) after a follow-up of 7±2 months post-hospital discharge^10^. Interestingly, we found that AKI phenotypes played a different role in the composite outcome of late kidney dysfunction, with community-acquired AKI, but not hospital-acquired AKI, being an independent risk factor.

The current study by de Andrade et al.^11^ sheds light on an area of knowledge that requires attention. Therefore, further research is still needed to better characterize renal long COVID-19 outcomes. The debate remains open and requires robust scientific evidence.
