# Supplementary material for: Renal long COVID-19: an ongoing debate requiring robust evidence
Source: J Bras Nefrol. 2025 Feb 24;47(1):e2025E001. doi: 10.1590/2175-8239-JBN-2025-E001en (PMC11864787; doi:10.1590/2175-8239-JBN-2025-E001en)
Supplement: Translation

# Translation: COVID-19 longa renal: um debate em andamento que requer evidências robustas

**DOI:** 10.1590/2175-8239-JBN-2025-E001pt

**Authors:** Heitor S. Ribeiro, Emmanuel A. Burdmann, Luis Yu

**Affiliations:** 1 Universidade de São Paulo, Faculdade de Medicina, Hospital das Clínicas HCFMUSP, Laboratório de Investigação Médica (LIM 12), São Paulo, SP, Brasil.

A COVID-19 tem um impacto devastador nos sistemas pulmonar e extrapulmonar. Os rins são frequentemente afetados durante a fase aguda da COVID-19, com aproximadamente 30% dos pacientes desenvolvendo injúria renal aguda (IRA) e quase metade evoluindo para óbito^1^. Para os sobreviventes de hospitalizações por COVID-19, recomenda-se o acompanhamento de longo prazo e a triagem de múltiplos órgãos devido ao impacto na morbidade e mortalidade a longo prazo, bem como ao possível ônus da COVID-19 longa para os sistemas de saúde^2^. A maior parte das evidências disponíveis sobre a COVID-19 longa tem se concentrado em desfechos cognitivos, neurológicos, psicológicos e pulmonares^3^. Poucos estudos de coorte prospectivos sobre a COVID-19 longa renal foram publicados, conforme detalhado na Tabela 1.

**Tabela 1:** Estudos de coorte prospectivos que investigam a COVID-19 longa renal

Autor e ano	Amostra (n e características)	Marcadores de função renal e pontos temporais	IRA durante a hospitalização	Desfechos da função renal no acompanhamento	Desenvolvimento e/ou progressão da DRC
Morin et al.^4^ 2021	478 pacientes hospitalizados	CrS 4 meses	20% (KDIGO)	2/177 pacientes apresentaram alteração persistente da função renal	NR
Li et al.^5^ 2022	155 pacientes hospitalizados	CrS 3, 12 e 24 meses proteinúria 24 meses	NR	A TFGe diminuiu entre 3 a 12 meses, mas não entre 12 a 24 meses Proteinúria 7% em 24 meses	NR
Gu et al.^6^ 2022	1734 pacientes hospitalizados	TFGe, proteinúria e hematúria 6 e 12 meses	6% (KDIGO)	sobreviventes com IRA apresentaram maior redução da função renal	NR
Huang et al.^7^ 2023	1733 pacientes hospitalizados	TFGe 6 meses	6% (KDIGO)	TFGe < 90 mL/min/1,73 m^2^: 479/1378 (35%)	NR
Schmidt-Lauber et al.^8^ 2023	443 casos não graves	TFGe, albuminúria, Dickkopf3, hematúria e piúria 9 meses	casos não graves (sem IRA)	TFGe média levemente menor em indivíduos pós-COVID-19 do que em indivíduos sem COVID-19	Desenvolvimento de DRC e albuminúria não diferiram entre pós-COVID-19 e não COVID-19
Mehrotra-Varma et al.^9^ 2024	748 pacientes hospitalizados e não hospitalizados com diabetes tipo 1	Códigos de diagnóstico CID-10 para DRC	NR	NR	Comparados aos pacientes sem COVID-19, pacientes hospitalizados (11,4% vs. 1,1%) e não hospitalizados (7,7% vs. 1,1%) apresentaram maior probabilidade de desenvolver DRC
Ribeiro et al.^10^ 2024	655 pacientes hospitalizados	TFGe, albuminúria, sedimento urinário anormal 7 meses	79%: 35% adquiridos na comunidade e 43% adquiridos no hospital (KDIGO)	16% desenvolveram baixa TFGe incidente (<60 mL/min/1,73m^2^) 27% apresentaram um declínio da TFGe de ≥25% 30% apresentaram albuminúria e 20% apresentaram sedimento urinário anormal	

Abreviações – IRA: injúria renal aguda; DRC: doença renal crônica; TFGe: taxa de filtração glomerular estimada; CID-10: Classificação Internacional de Doenças, Décima Revisão; KDIGO: Kidney Disease Improving Global Outcomes; TRS: terapia renal substitutiva; MAKE: eventos renais adversos maiores; NR: não relatado; CrS: creatinina sérica.

Nesta edição do Jornal Brasileiro de Nefrologia, de Andrade et al.^11^ avaliaram prospectivamente 360 pacientes graves com COVID-19 e IRA, durante a fase aguda da doença, que necessitaram de consulta com um nefrologista em um hospital terciário na cidade de Porto Alegre, RS. Os autores definiram a IRA de acordo com os critérios do *Kidney Disease: Improving Global Outcomes* (KDIGO), com base na creatinina sérica (CrS). Eles também avaliaram a albuminúria e a necessidade de terapia renal substitutiva (TRS). A recuperação da função renal foi definida como a independência da TRS ou o retorno da CrS a um valor dentro de 50% do basal, de acordo com a *Acute Dialysis Quality Initiative*. De modo geral, os autores constataram uma frequência muito alta de IRA grave (KDIGO III - 90,8%), na qual 90% necessitaram de TRS, resultando em uma elevada taxa de mortalidade em 90 dias (88%, com apenas 10% dos pacientes recuperando a função renal). Após um ano de acompanhamento, a taxa de mortalidade também foi bastante elevada (89%). Entre os sobreviventes, a taxa de filtração glomerular estimada (TFGe) após um ano foi inferior aos níveis basais. Uma questão metodológica deste estudo foi a definição proposta do valor basal de CrS para o diagnóstico de IRA, na qual os autores analisaram o valor médio de creatinina entre 7 e 365 dias antes da hospitalização. Entretanto, caso essa informação não estivesse disponível, foi adotado o valor mínimo de CrS durante a hospitalização. Essa variação na definição pode enviesar a validade interna do diagnóstico de IRA, especialmente porque os autores não informam a frequência de pacientes sem valores basais de CrS. Ao adotar o valor mínimo de CrS durante a hospitalização, os autores podem ter superestimado o diagnóstico de IRA. Além disso, a perda de massa muscular, comumente observada em pacientes críticos com COVID-19, pode ter levado a uma redução nos níveis de CrS durante a hospitalização^12^, o que pode ter resultado em um valor de CrS inferior ao valor basal real.

Outro achado que merece consideração é a frequência extremamente elevada de IRA grave, raramente reportada na literatura. Corroborando esse achado, 98% dos pacientes necessitaram de ventilação mecânica e a maioria deles precisou de TRS (90%). Conforme esperado, uma consulta de nefrologia no ambiente crítico geralmente é solicitada para os casos mais graves, especialmente em uma situação de pandemia, resultando na seleção de casos graves de IRA. Não obstante, foi relatado que a consulta de nefrologia para indivíduos com COVID-19 geralmente resulta em maior sobrevida do paciente^13,14^. Portanto, houve um claro viés de seleção da amostra, o qual limita a generalização para pacientes com COVID-19 não grave e pacientes com estágios iniciais de IRA que geralmente não são encaminhados aos nefrologistas.

Os desfechos primários foram mortalidade em 90 dias, recuperação da função renal e dependência de TRS. No entanto, os autores não relataram o tempo médio de sobrevida. Ao final do período de 90 dias, apenas 12% dos pacientes haviam sobrevivido. A suposição de um período de acompanhamento de 90 dias pode levar à expectativa de um acompanhamento de longo prazo após a alta hospitalar, o que não ocorreu para a maioria dos pacientes. De fato, a mortalidade aos 30 dias foi de 66% e aos 60 dias foi de 85%, semelhante à taxa de mortalidade aos 90 dias. Portanto, esses achados devem ser cuidadosamente interpretados como um desfecho intra-hospitalar. Além disso, os autores incluíram um acompanhamento de um ano para uma amostra muito pequena de sobreviventes (n = 43; 12%), na qual somente 25 dos pacientes remanescentes (58%) tinham medições de TFGe disponíveis. Eles demonstraram que a TFGe dos sobreviventes foi inferior à TFGe basal (65,9 *vs.* 85,5 mL/min/1,73 m^2^). Embora a análise tenha sido secundária e exploratória, deve-se ter cautela ao extrapolar os achados para os sobreviventes da COVID-19, uma vez que a amostra do estudo foi derivada de uma coorte tendenciosa, com mortalidade hospitalar muito elevada, resultando em um longo acompanhamento de uma amostra muito pequena.

Embora o estudo seja inovador ao recrutar uma amostra de pacientes que necessitaram de consulta nefrológica, já existem dados suficientes na literatura demonstrando o impacto da COVID-19 nos desfechos renais durante o período hospitalar^1^. No momento, a discussão deve concentrar-se em determinar se a COVID-19 longa afeta os rins, em vez de como os rins são um órgão-alvo durante a fase aguda da infecção. Conforme demonstrado na Tabela 1, atualmente existem poucos estudos disponíveis abordando essa importante questão. Nosso grupo de pesquisa está realizando um estudo de coorte prospectivo com mais de 650 sobreviventes da hospitalização por COVID-19 na cidade de São Paulo, SP^15^, com acompanhamento anual por até cinco anos após a alta hospitalar. Dados preliminares apresentados no XXXII Congresso Brasileiro de Nefrologia e na *ASN Kidney Week 2024* mostraram que 27% dos pacientes apresentaram um declínio na TFGe ≥25%, e 16% tiveram incidência de TFGe baixa (<60 mL/min/1,73 m^2^) após um acompanhamento de 7±2 meses pós-alta hospitalar^10^. Curiosamente, constatamos que os fenótipos de IRA desempenharam um papel diferente no desfecho composto da disfunção renal tardia, sendo a IRA adquirida na comunidade, e não a IRA adquirida no hospital, um fator de risco independente.

O estudo atual por de Andrade et al.^11^ lança luz sobre uma área de conhecimento que requer atenção. Portanto, ainda são necessárias mais pesquisas para melhor caracterizar os desfechos da COVID-19 longa renal. O debate permanece aberto e demanda evidências científicas robustas.
